# Direct atomic scale determination of magnetic ion partition in a room temperature multiferroic material

**DOI:** 10.1038/s41598-017-01902-1

**Published:** 2017-05-11

**Authors:** Lynette Keeney, Clive Downing, Michael Schmidt, Martyn E. Pemble, Valeria Nicolosi, Roger W. Whatmore

**Affiliations:** 1 Tyndall National Institute, University College Cork, ‘Lee Maltings’, Dyke Parade, Cork, Ireland; 2 School of Chemistry, CRANN, AMBER, Trinity College Dublin, Dublin 2, Ireland; 3 Department of Chemistry, University College Cork, Cork, Ireland; 4 Department of Materials, Faculty of Engineering, Imperial College London, London, SW7 2AZ United Kingdom

## Abstract

The five-layer Aurivillius phase Bi_6_Ti_x_Fe_y_Mn_z_O_18_ system is a rare example of a single-phase room temperature multiferroic material. To optimise its properties and exploit it for future memory storage applications, it is necessary to understand the origin of the room temperature magnetisation. In this work we use high resolution scanning transmission electron microscopy, EDX and EELS to discover how closely-packed Ti/Mn/Fe cations of similar atomic number are arranged, both within the perfect structure and within defect regions. Direct evidence for partitioning of the magnetic cations (Mn and Fe) to the central three of the five perovskite (PK) layers is presented, which reveals a marked preference for Mn to partition to the central layer. We infer this is most probably due to elastic strain energy considerations. The observed increase (>8%) in magnetic cation content at the central PK layers engenders up to a 90% increase in potential ferromagnetic spin alignments in the central layer and this could be significant in terms of creating pathways to the long-range room temperature magnetic order observed in this distinct and intriguing material system.

## Introduction

Multi-state memory devices based on single-phase multiferroic materials (containing both ferroelectric (FE) and ferromagnetic (FM) memory states) have been road-mapped^1^ as promising architectures for memory scaling beyond current technologies. However, there are relatively few^2–5^ materials demonstrating FE and FM properties in a single-phase at room temperature (RT), and consequently no such devices currently exist.

Due to conflicting electronic structure requirements for ferroelectricity and ferromagnetism it has been considered for some time that the two properties tend to be mutually exclusive^6^. However, recently there has been considerable interest in the engineering of layer-structured materials to accommodate both FE and FM cations within the same structure^4, 5, 7^. The Aurivillius (Bi_2_O_2_(*A*_m−1_ *B*_m_O_3m+1_))^8^ FEs offer a naturally layer structured system in which layers of m perovskite (PK) blocks are sandwiched between (Bi_2_O_2_)^2+^ layers with a structure that approximates that of fluorite. The PK layers readily accommodate magnetic cations (Fe, Mn, Co) on the PK B-sites, and compositions in the system Bi_m+1_Fe_m−3_Ti_3_O_3m+3_ (BTFO) have been shown to exist with values of m ranging from 4 to 9^9^ with evidence of antiferromagnetic, weak ferromagnetic and magnetoelectric behaviour^10–12^. Recently, evidence has been reported for exchange-bias and spin cluster glass effects, together with weak ferromagnetism at ca100K in the m = 9 BTFO system^13^. The inclusion of other magnetic ions such as Co or Mn on the B-site, together with Fe has been pursued with the intention of improving the magnetic properties of the BTFO system, and strong FM and FE has been reported in m = 4 Bi_5_Fe_0.5_Co_0.5_Ti_3_O_15_^14^. However, it is now well-established that very small (<0.1%) amounts of FM impurities such as CoFe_2_O_4_ can be seriously misleading when seeking FM behaviour in these systems^15^. FE/FM multiferroicity was demonstrated in the single-phase Aurivillius system: Bi_6_Ti_x_Fe_y_Mn_z_O_18_ (B6TFMO) where x = 2.80–3.04, y = 1.32–1.52, z = 0.54–0.64^5, 15, 16^. Thin films of these materials possess spontaneous polarisation (e.g. Ps~30 μC/cm^2^ in m = 3 BTFO)^17^ and saturation magnetisation (M_S;_ up to 215 emu/cm^3^ in B6TFMO)^16^ and demonstrate magnetic-field-induced FE domain switching at RT. This is the only material to date in which the multiferroicity has been demonstrated to originate from the Aurivillius phase at a defined confidence level (>99.5%)^15^. The B6TFMO system is therefore an exciting candidate for potential use in multiferroic, magnetoelectric devices which could potentially meet future industry requirements in multi-state memory applications^18, 19^.

**Figure 1:**
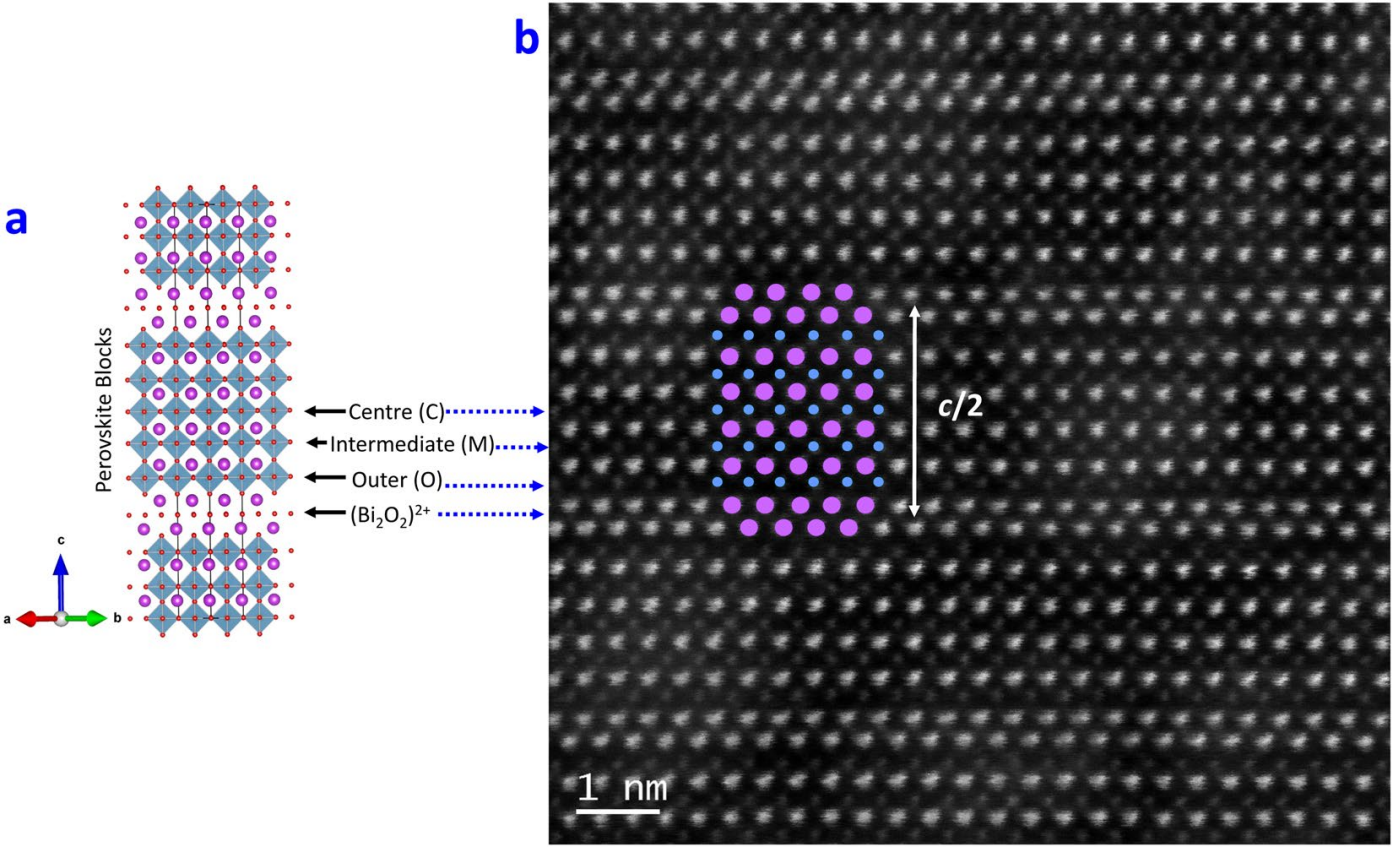
(**a**) Schematic of a five-layered Aurivillius phase structure^19^, illustrating layered intergrowths of fluorite-like [Bi_2_O_2_]^2+^ units alternating with perovskite-like units having Bi cations at the A-sites (purple) and transition metal cations at the B-sites. BO_6_ octahedra are displayed in blue and oxygen atoms are displayed in red. (**b**) Atomic resolution HAADF-STEM image for B6TFMO. Bi cations are represented by purple circles and transition metals cations (Ti/Mn/Fe) are represented by blue circles.

RT FE/FM multiferroicity in B6TFMO and the other similar compounds is unexpected, given the relatively dilute level of magnetic cations available to undergo FM exchange interactions. There are five PK layers per half-unit cell (*m* = 5) in this structure, giving three symmetrically-distinct *B*-site locations over which the magnetic cations (Mn and Fe) can be distributed^20^ (Fig. 1(a))^21^. Our knowledge to-date on magnetic cation partitioning^9, 22–25^ has mainly depended on inference from structural refinement and statistical methods, which cannot provide information on the localisation of cations for example around defect sites. Previous reports of magnetic cation partitioning within the perovskite (PK) layers in Aurivillius structures, were determined by the Rietveld refinement of X-ray and neutron powder diffraction data. In the *m* = 3 compound Bi_2_Sr_2_Nb_2_MnO_12−δ_, the Mn ions were observed to order into the centre layer, away from the (Bi_2_O_2_)^2+^ layers^22^. In Bi_5_Ti_3_CrO_15_ (*m* = 4), Giddings *et al*.^23^ presented evidence from Reitveld analysis of neutron diffraction data that the Cr ions partition partially into the central two layers. Lomanova *et al*.^9^ used Mössbauer spectroscopy to study the BTFO system for 3.5 ≤ m ≤ 9, and showed that for m < 7, the Fe partitions away from the PK blocks adjacent to the (Bi_2_O_2_)^2+^ layers, but that for m ≥ 7 the Fe and Ti are randomly distributed over the PK B-sites. However, Hevoches *et al*.^24^ were unable to detect any departure from a random distribution of Fe ions in the compound Bi_5_Ti_3_FeO_15_, by either Rietveld refinement of neutron diffraction data, or Mossbauer spectroscopy. By contrast, partial-partitioning of the Fe away from the PK blocks adjacent to the (Bi_2_O_2_)^2+^ layers, and into the central three PK layers was observed by Rietveld refinement of X-ray diffraction data in the *m* = 5 compound Bi_6_Ti_3_Fe_2_O_18_^20^. Statistical analysis of extended X-ray absorption fine structure (EXAFS) data for this compound has also indicated a preference for the Fe^3+^ cations to occupy the ‘inner’ sites within the PK layers^25^. However, EXAFS is a statistical technique whereby experimental information on unique octahedral sites is refined to theoretically calculated structural models, and no information can be derived on the extent of cation partitioning. Recently, there has been evidence of Fe^3+^ ion partitioning presented by Huang *et al*.^13^ from HAADF-EELS analysis in the m = 9 BTFO system. Here, Fe^3+^ was observed to partition away from the central layers of the PK blocks and towards the (Bi_2_O_2_)^2+^ layers. This is an interesting result which contrasts with the earlier work^9^ that indicated a tendency for the Fe to partition into the centres of the PK blocks layers or, for compositions with m ≥ 7, to be randomly distributed. Huang *et al*.^13^ do not remark upon this dispartity between their observations and the earlier work, but make the important observation that the structural partitioning of magnetic cations may be implicated in the magnetic behaviour of this type of material. We can note that the disparity between different authors’ observations of Fe^3+^ cation partitioning in different BTFO systems may be a consequence of differences between sample preparation methodologies. Prior to the work by Huang *et al*.^13^, while there had been studies at atomic resolution of PK multi-layer structures^26^, PK BiFeO_3_ layers^27^ and Ruddelsden-Popper faults in perovskite PK BaSnO_3_ layers^28^, there had been no direct observations of *B*-site cation distributions within Aurivillius phase structures, either generally or specifically in the multiferroic B6TFMO system^29, 30^. As Huang *et al*.^13^ have observed, information on magnetic cation distributions is important to further our understanding of multiferroic systems. Nearest neighbour (NN) and next-nearest-neighbour (NNN) interactions between magnetic cations are key in determining the occurrence of ferromagnetism and antiferromagnetism in oxides. For the Bi_5_Ti_3_FeO_15_ (*m* = 4 BTFO) system, density functional theory (DFT) predicts rather strong couplings (J_NN_∼40–50 meV) for Fe^3+^ cations in NN positions^31^. However for Fe^3+^ cations distributed in NNN positions, the coupling is relatively weak (J_NNN_ ∼ 1–2 meV) and becomes negligible among second-nearest neighbours. The juxtaposition and arrangement of Mn-O-Fe and Mn-O-Mn linkages is important in determining the existence of ferromagnetism, or antiferromagnetism, depending on the valence states of the ions concerned and the bond lengths^32–34^. Therefore, as both Birenbaum and Ederer^31^ and Huang *et al*.^13^ have pointed-out, knowledge of the *B*-site distribution of the magnetic cations in the Aurivillius multiferroics in general is important in understanding their ferromagnetism. We consider here specifically the distribution of Mn/Fe cations in B6TFMO for the same reason.

Here we use atomic-resolution aberration-corrected high-angle annular dark-field scanning transmission electron microscopy (HAADF-STEM) combined with EDX (energy dispersive X-ray analysis) and EELS (electron energy loss spectroscopy) analytical techniques to differentiate between closely-packed cations with similar atomic number (*Z*) and distinguish Ti/Mn/Fe cations dominating at certain *B*-site locations. We obtain direct atomic-scale visualisation of magnetic cation partitioning and demonstrate a clear preference for Mn cations to partition into the central PK layer, which we infer is due to strain- and electrostatic-energy considerations. We reveal further changes in the relative proportions of magnetic ions in the region of stacking fault defects and Out-of-Phase Boundaries (OPBs). We believe this evidence to be central in explaining pathways to long-range magnetic order and the distinct RT multiferroic properties of this tantalising material system.

## Results

### Atomic Resolution Imaging and Chemical Mapping

**Figure 2:**
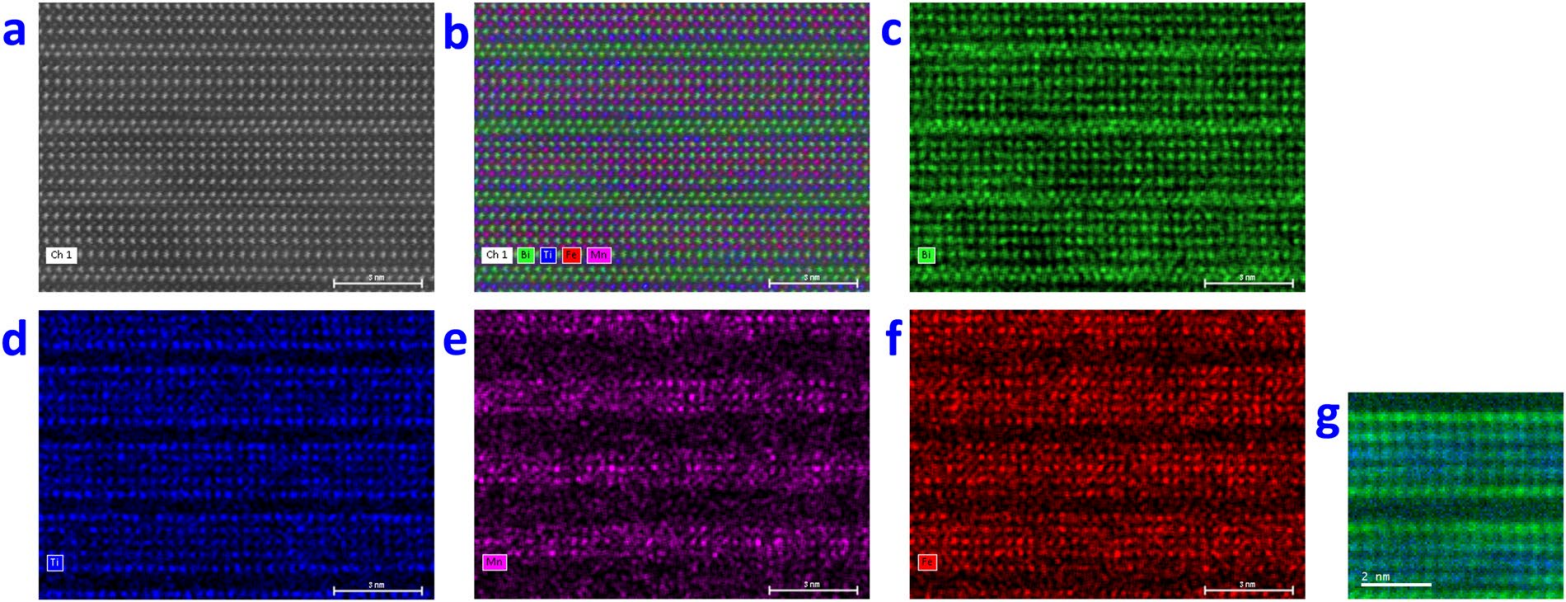
Representative atomic resolution chemical mapping images for B6TFMO-A in a region of the crystallite without out-of-phase boundaries (OPBs)/stacking faults. (**a**) Annular dark-field image and (**b** to **f**) corresponding EDX chemical composition maps: (**b**) overlay map showing annular dark-field image (white), Bi (green), Ti (blue), Mn (pink), Fe (red), (**c**) Bi map, (**d**) Ti map, (**e**) Mn map, (**f**) Fe map. (**g**) EELS chemical composition maps of a different region of B6TFMO-A (refer to Supplementary Figure S3(a) for corresponding HAADF-STEM image) with Ti in green and Mn in blue.

**Figure 3:**
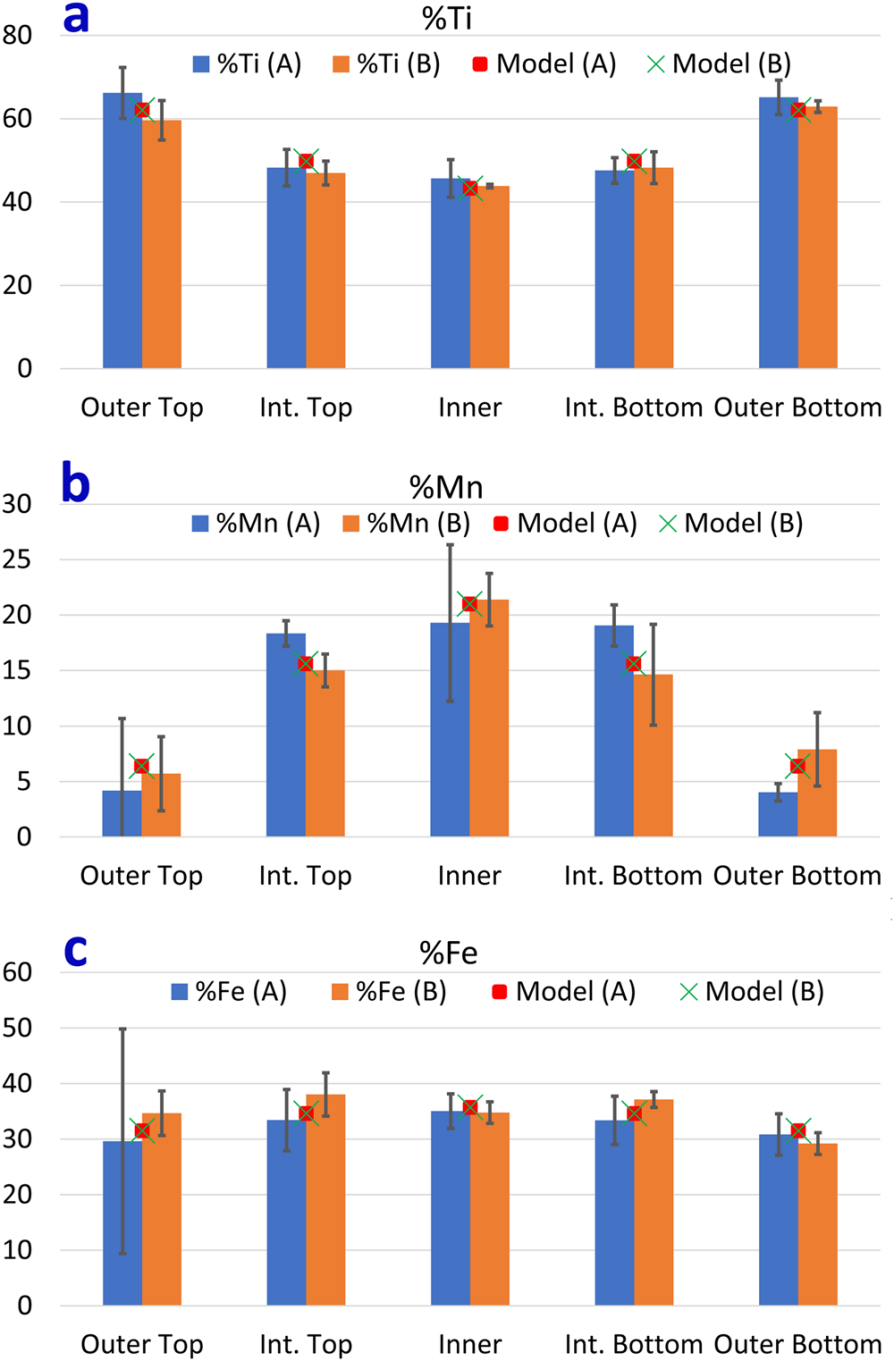
The relative B-site proportions (in %) of Ti, Mn and Fe were quantified by taking horizontal line profiles of the EDX data across the B-sites of the PK layers over three half-unit cells analysed and the averages for the outer top (O-top), intermediate top (M-top), centre (C), intermediate bottom (M-bottom) and outer bottom (O-bottom) PK layers. These are represented in the histograms shown in (**a** to **c**), respectively. Note that the data presented is for regions without OPBs/stacking faults. The error bars represent the spread in measured compositions for three PK layers. The experimental values of C(x) (Supplementary Table S1) have been fitted using Equations (1) and (2) and the modelled values are plotted for both B6TFMO-A and B6TFMO-B as represented by the Model (A) red square and the Model (B) green cross.

Two samples, B6TFMO-A and B6TFMO-B, of similar composition, but from different growth runs, were selected for imaging experiments. Both samples are FE and FM at RT with M_S_ values up to 215 emu.cm^−3^ ^5, 16^. By sampling through the depth of the B6TFMO samples along a column of atoms, atomic-resolution HAADF-STEM images of differing magnification were collected (Fig. 1(b) and Supplementary Figure S1). Based on intensity and cation radii differences, the heavier (*Z* = 83) and larger (radius ≥ 1.11 Å)^35^ Bi cations at the fluorite-type layers and at the *A*-sites of the PK layers can clearly be distinguished from the transition metal cations at the *B*-sites of the PK layers. This data, in conjunction with X-ray diffraction and transmission electron microscopy (TEM) images^5, 15, 16^, confirms that the majority of the samples are *m* = 5. By uncorrected STEM-EDX/EELS mapping, it is impossible to distinguish between Ti/Mn/Fe (*Z* = 22, 25 and 26, respectively) cations in a closely-packed material at atomic-scale. The NION is equipped with a 100 mm^2^ windowless EDX detector combined with a probe corrected cold field emission electron source which allows atomic-resolution mapping with high sensitivity, making it possible to chemically identify not only the Bi cations but also between the Ti, Mn and Fe cations dominating at *B*-site locations in B6TFMO. Notably, from the Ti maps in both samples (Fig. 2(d)) and Supplementary Figure S2(d)), we observe an increased signal for Ti within the columns of atoms at the outer (*O*) PK layers, compared with the intermediate (*M*) and centre (*C*) PK layers. On the other hand, we observe a striking decrease in signal for Mn within the columns of atoms at the *O* PK layers and note an obvious preference for Mn to partition into the *M* and *C* PK layers (Fig. 2(e) and Supplementary Figure S2(e)). Atomic-resolution EELS images (Fig. 2(g) and Supplementary Figure S2(g)) confirm these observations. The average relative *B*-site proportions of Ti, Mn and Fe at the *O*, *M* and *C* layers of samples B6TFMO-A and B6TFMO-B are presented in Supplementary Table S1 and in the histograms in Fig. 3. The *B*-site proportion (expressed in terms of percentage of *B*-site occupancy) of Ti decreases considerably (approx. 20%) from the *O* layers to the *C* layers. From the spread in the measured proportions made by averaging along 12 nm lengths of four separate PK layers of each type in Sample A and 6 nm lengths of three separate PK layers of each type in Sample B (see the Methods section below), and from the two (unrelated) samples A and B, we estimate the uncertainty in these proportions as being ca 3%. There is a significant (approx. 17 to 20%) increase in the *B*-site proportion of Mn from the *O* layers to the *C* layers. Overall, there appears to be a slight preference (3 to 5% increase) for Fe to partition into the inner PK sites rather than the O sites, but this preference is not significant. The preference for Mn to partition into the *C* PK sites and for Ti to partition to the *O* PK sites leads to a significant (8 to 9%) increase in the proportion of magnetic cations at the *C* layer sites compared with a fully a disordered distribution of *B*-site cations.

**Figure 4:**
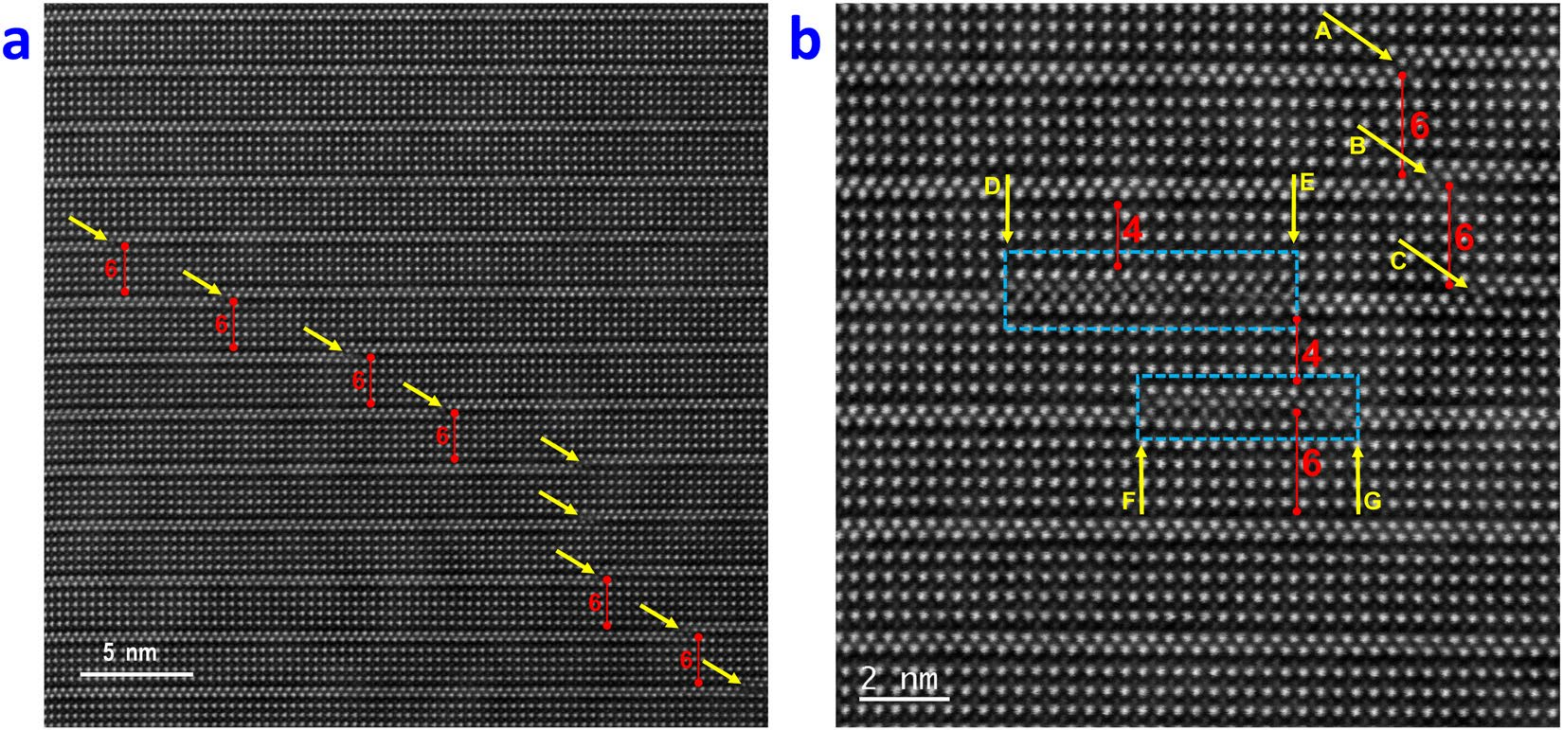
Representative atomic resolution images of B6TFMO in a region of the crystallite displaying OPBs/stacking faults. HAADF-STEM images of **(a)** lower magnification for B6TFMO-A and **(b)** higher magnification for B6TFMO-B. The numbers displayed in red designate the number of PK layers (m) in the region designated by the red bar. The majority of the sample has an m = 5 structure. Yellow arrows demonstrate the position of the OPBs and blue rectangles indicate stacking fault regions.

**Figure 5:**
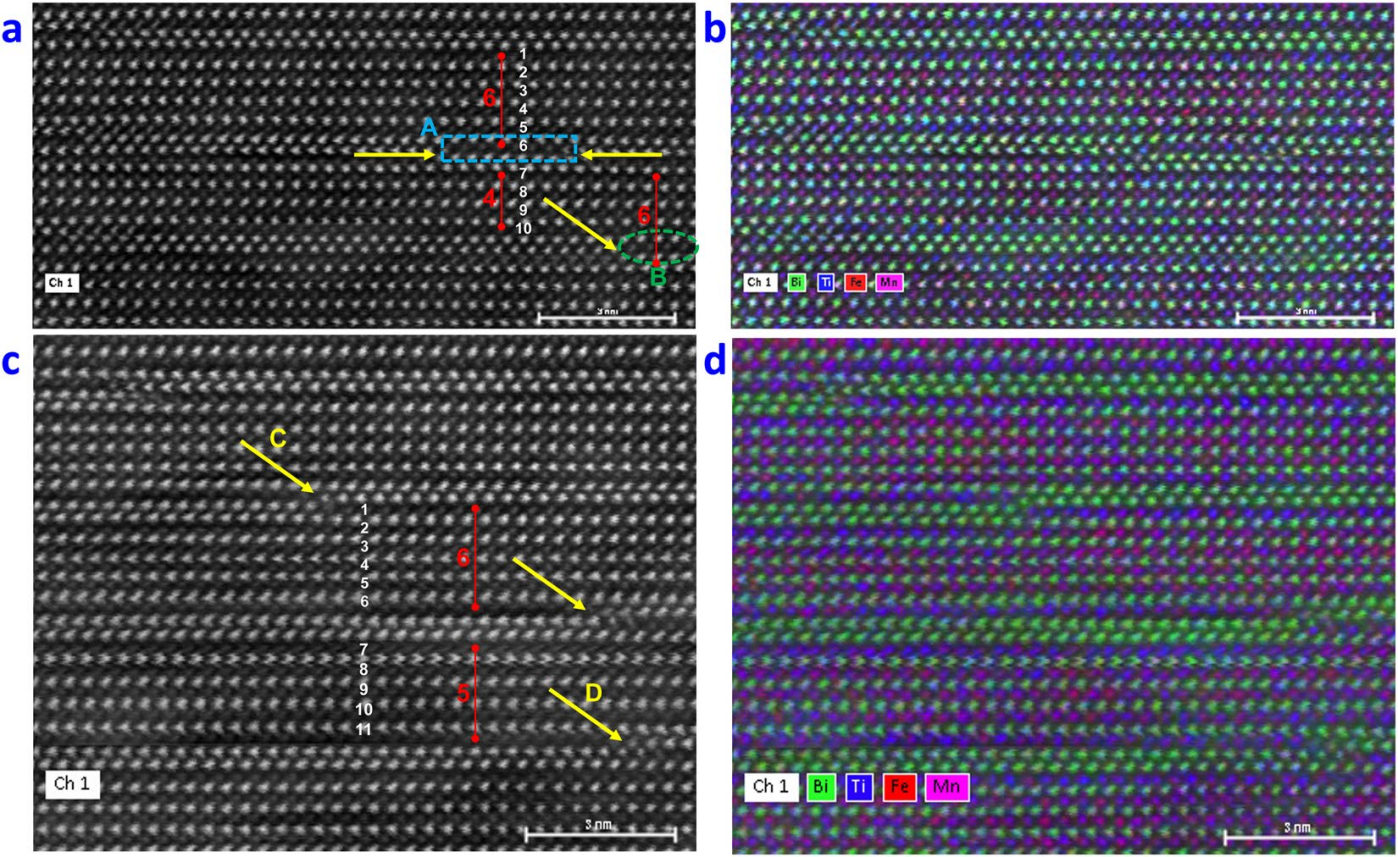
Representative atomic resolution EDX images for B6TFMO-B ((**a** and **b**)) and B6TFMO-A ((**c** and **d**)) in a region of the crystallite displaying OPBs/stacking faults. (**a** and **c**) annular dark-field images and (**b** and **d**) corresponding EDX chemical composition overlay maps showing annular dark-field image (white), Bi (green), Ti (blue), Mn (pink), Fe (red). Yellow arrows demonstrate the positions of the OPBs, the blue rectangle A signifies a stacking fault region and the green ellipsoid B designates a region in the vicinity of an OPB. The numbers displayed in red designate the number of PK layers (*m*) in the region designated by the red bar. The majority of the sample has an *m* = 5 structure. PK layers used for quantification are labelled by the numbers in white. The relative B-site proportions of Ti, Mn and Fe were quantified by taking horizontal line profiles of the EDX data across regions indicated and are tabulated in Tables 1 and 2.

Some regions of the samples display OPB defects where there is displacement by a fraction of a lattice parameter (*c*/*x)* between two adjacent B6TFMO layers parallel to the *z*-direction. These are a common occurrence in materials of high structural anisotropy, such as the Aurivillius phases, although the type and density of these defects can vary widely from sample to sample and from grain to grain within samples^36, 37^. The OPB density for B6TFMO-A and B6TFMO-B is not high enough in this case to cause peak splitting in conventional X-ray diffraction analysis^16, 36^; but the atomic structure of the OPBs can easily be seen at the local scale by this optimised HAADF-STEM technique and appear as steps as viewed edge-on in Fig. 4. These can form when the *c*-axis is inclined with respect to the substrate surface allowing propagation of OPBs through the film, as delineated by the arrows in Fig. 4(a) or when adjacent, out-of-phase nuclei meet during growth as indicated by arrows A/B/C in Fig. 4(b) ^37^. OPBs can be accompanied by an insertion of an ‘extra’ perovskite block, giving regions of different *m*-layers, as evident by *m* = 6 regions in both figures. Stacking faults can also be visible in Fig. 4(b) (highlighted by blue rectangles), where there are sub-unit-cell intergrowths of different *m*-layers (e.g. *m* = 6, *m* = 4) and can terminate in an OPB (arrows D/E/G). It is obvious from these images that physical interruption of the lattice is compensated by a deficiency or excess of bismuth atoms. This is further demonstrated by atomic-resolution EDX (Fig. 5, and the data in Tables 1 and 2) and EELS (Figure S3(i–p)). Atomic-resolution chemical mapping of a stacking fault region (blue rectangle A in Fig. 5(a)) with *m* = 6 and *m* = 4 intergrowths reveals that Ti content is increased and Mn content is drastically decreased locally at the defect (Layer 6/region A in Fig. 5(a)) compared with the defect-free average (Supplementary Table S1). The overall effect of the stacking fault on the average proportion of Mn in layers 1–10 (Fig. 5(a and c) and Tables 1 and 2) is only a ~2% decrease compared with the defect-free average, which is not significant.

**Table 1:** The relative B-site proportions of Ti, Mn and Fe quantified by taking horizontal line profiles of the EDX data across regions indicated in Fig. 5(a).

B6TFMO-B Region	% Ti	% Mn	% Fe	% Magnetic Cations
Layer 1	62	3	35	37
Layer 2	46	11	43	54
Layer 3	38	26	36	62
Layer 4	48	13	39	52
Layer 5	65	8	27	35
Layer 6 (A)	65	0	35	35
***Average of 6 layers***	***54***	***10***	***36***	***46***
Layer 7	49	18	32	51
Layer 8	45	13	42	55
Layer 9	47	10	43	53
Layer 10	70	4	26	30
***Average of 4 layers***	***53***	***11***	***36***	***47***
**B**	82	0	18	18

**Table 2:** The relative B-site proportions of Ti, Mn and Fe quantified by taking horizontal line profiles of the EDX data across regions indicated in Fig. 5(c).

B6TFMO-A Region	% Ti	% Mn	% Fe	% Magnetic Cations
Layer 1	67	2	31	33
Layer 2	54	15	31	46
Layer 3	41	20	39	59
Layer 4	36	24	40	64
Layer 5	41	14	45	59
Layer 6	62	10	29	38
***Average of 6 layers***	***50***	***14***	***36***	***50***
Layer 7	65	7	28	35
Layer 8	43	19	37	56
Layer 9	36	23	41	64
Layer 10	47	17	35	53
Layer 11	63	5	32	37
***Average of 5 layers***	***51***	***14***	***35***	***49***

The considerable effect that an OPB has on the local stoichiometry in the vicinity of the OPB is demonstrated by the green ellipsoid B in Fig. 5(a) and the EDX data in Table 1 (region B). A substantial increase (~30%) in Ti compensates for the deficiency in Bi, while the Fe concentration is depleted (~17%) and there is virtually no Mn present (Fig. 5(a,b), Table 1) at the OPB defect region. On the other hand, the presence of OPBs does not have a significant effect on the larger regions. For example in layers 1–11 in the region between OPBs C and D in Fig. 5(c), the amounts of Ti, Fe and Mn are not changed significantly (Table 2) compared with the defect-free average (Supplementary Table S1). It is important to note that there is a significant ~13% (~8%Mn and ~5%Fe) increase in magnetic cation partitioning to the *C* PK layers (layers 3/4/9) for this region between OPBs, compared with a ~9% (~6%Mn and ~3%Fe) increase in partitioning to the *C* PK layers for defect-free regions. It therefore appears that disruptions of the Aurivillius phase lattice by OPBs may result in further partitioning of magnetic cations to the *C* PK layers of B6TFMO.

OPBs and structural defects can have considerable impact upon material properties^38^. Changes in local chemistry at the defects and corresponding changes to magnetic cation distribution in the defect regions could be significant in determining magnetic behaviour in B6TFMO and its sample-to-sample variability. As seen in this work and in previous TEM studies^16^, the defects vary strongly from grain-to-grain and even vary from region-to-region within the same grain. We have also seen that the extent of magnetoelectric switching varies from sample-to-sample and from grain-to-grain in B6TFMO^5, 16^. Correlating magnetic and magnetoelectric properties with structural defects is the subject of on-going/future work.

## Discussion

### Mechanisms driving cation partitioning in the BT6FMO Structure

The observation that Mn partitions preferentially into the *C* layers of the 5-layer Aurivillius layer structure of B6TFMO is extremely interesting in terms of its implications for magnetic properties and has not been directly observed before. The chemical vapour deposition technique used to synthesise the B6TFMO samples^16^ does not impose any layer-by-layer ordering of the precursors during the deposition process, which must therefore occur as a natural part of the fabrication process. There are three possible mechanisms for cation partitioning of the cations.

Garcia-Guaderrama *et al*. start with Kikuchi’s model^39^ for Aurivillius phase materials. This model, which follows Armstrong and Newnham^40^, uses ionic radius arguments to deduce that the PK blocks are under compression, and the (Bi_2_O_2_)^2+^ layers under tension, providing a strain energy to drive cations larger than Ti^4+^ away from the layers closest to the (Bi_2_O_2_)^2+^ layers. Giddings *et al*.^23^ propose two reasons for the partitioning of Cr^3+^ way from the outer layers in the *m* = 4 Bi_5_Ti_3_CrO_15_. The first is the preference for Ti^4+^ to occupy more-distorted octahedra (a reason also proposed by Lomanova *et al*.^9^ in an analysis of several different Bi_4_Ti_3_O_12_−BiFeO_3_ compounds) with the degree of octahedral distortion determined by the Shannon site distortion parameter^41^ Δ_n_ (see Supplementary Information). The second is based upon Madelung energy calculations indicating an electrostatic preference for the more-highly-charged Ti^4+^ cation to partition close to the outer layer of oxygens in the (Bi_2_O_2_)^2+^ layers. Thus the three potential mechanisms to drive magnetic cation partitioning are: strain-energy considerations, the preference of Ti^4+^ to occupy more-highly-distorted octahedra and electrostatic energy considerations.

Calculation of Δ_n_ values for RT B6TFMO based on the previously-published structure^20^ indicates values of 0.70, 0.46 and 0.05 respectively for the *O*, *M* and *C* layer octahedra (Fig. 1(a)). These are much smaller than the values reported for Bi_5_Ti_3_CrO_15_, and would be further reduced at the synthesis temperature, although the *M* and *C* sites would probably become more similar at higher temperatures^23^. These results indicate that site asymmetry is not a strong driver for cation partitioning in B6TFMO, leaving strain energy considerations^39^ and electrostatic interactions as being the most likely mechanisms. Of these, the calculations of Birenbaum and Ederer^31, 42^ indicate that the strain energy factor is more important.

Consider the high-spin^31^ ionic radii (in 6-fold coordination) for the *B*-site ions in the B6TFMO system (Supplementary Table S2)^34^. The ionic radii for Fe^3+^ and Mn^3+^ are both slightly bigger than Ti^4+^. According to Kikuchi^39^, that would drive these ions away from (Bi_2_O_2_)^2+^, and as both are trivalent, the electrostatic argument for partitioning would also apply. However, the ionic radii for Fe^3+^ and Mn^3+^ are very similar (and the charges are identical), so these mechanisms by themselves would not explain why Mn is preferentially pushed into the *C* PK blocks. However, Mn^2+^ is much bigger than both Ti^4+^ and Fe^3+^.

We know (see Supplementary Information) that it is energetically much easier to reduce Mn^3+^ to Mn^2+^ than Fe^3+^ to Fe^2+^ at the B6TFMO synthesis temperature (1100 K), so a substantial part of the Mn in the system will be present as Mn^2+^. Mn^2+^ is 33% bigger than Ti^4+^, but Fe^3+^ is only 6% bigger than Ti^4+^, so there is a much larger strain-energy (Kikuchi)^39^ impetus towards partitioning of Mn^2+^ than Fe^3+^. Similarly, we would expect that the divalent Mn^2+^ would have a stronger preference than Fe^3+^ for partitioning away from the outer oxygens of the (Bi_2_O_2_)^2+^ layers. On the other hand Fe^3+^ would still, as a trivalent ion slightly bigger than Ti^4+^, have some drive for partitioning. This would explain our observation of a 15% increase in the *B*-site proportion of Mn at the *C* layers, compared with only a 3–5% increase in Fe partitioning to the *C* layers. This only slight preference for Fe to partition into the *C* sites is consistent with Mössbauer data^9^ and DFT calculations for the 4-layered Bi_5_Ti_3_FeO_15_^31^.

We can use the Boltzmann equation (see Supplementary Information) to model the expected distribution of the Mn and Fe within the three PK layers:

1 $$C(x)=C(0)\exp \{\frac{-b{x}^{2}}{2\sigma }\}$$

Which is a Gaussian function, with

2 $$\sigma =\sqrt{\frac{kT}{2b}},{\rm{or}}\,{b}=\frac{kT}{2{\sigma }^{2}}$$

The experimental values of C(x) (Supplementary Table S1) have been fitted using this equation and the modelled values are plotted for both B6TFMO-A and B6TFMO-B in Fig. 3. It can be seen that the fit is a good one, and the two values of σ are 0.51 ± 0.01α and 1.6 ± 0.1α for Mn and Fe respectively, corresponding to the values of *b* being 0.182 eV/nm^2^ and 0.0185 eV/nm^2^ for Mn and Fe respectively. This means that the energy difference between the three available sites varies ten times faster for manganese relative to iron. The energy differences between the *C*-to-*M* and *C*-to-*O* PK *B*-sites are given in Table 3.

**Table 3:** Energy difference variation between centre-to-intermediate and centre-to-outer B6TFMO B-sites for Mn and Fe.

B-Site difference	Mn	Fe
centre-to-intermediate (eV)	0.029	0.003
centre-to-outer (eV)	0.117	0.012

As the temperature is reduced during sample cooling, we would expect the Gibbs free energy ΔG for Mn reduction to become positive at approximately 1060 K. Below this temperature, Mn^2+^ will probably start to convert back to Mn^3+^ in the oxidising atmosphere. Hitherto there has been no direct determination of the oxidation state of Mn in the Aurivillius bismuth titanates, although it has been inferred from detailed stoichiometry studies^5^ that there are variable amounts of Mn^3+^ and Mn^4+^ in different grains of B6TFO thin films. In pure perovskites such as lead zirconate titanate^43, 44^ and barium titanate^45, 46^ ceramics it has been shown by Electron Paramagnetic Resonance (EPR) that Mn exists as Mn^2+^, Mn^3+^ and Mn^4+^, depending on the partial pressure of the oxygen in the firing atmosphere, with a tendency to form Mn^4+^ as the atmosphere becomes more oxidising, or a tendency to form Mn^2+^ in the presence of donor dopants such as La^3+^ or Nb^5+^. In Mn-doped Pb(Mg_0.33_Nb_0.67_)O_3_-PbTiO_3_ single crystals, the Mn has been shown by X-ray Absorption Spectroscopy^47^ to be mainly present as Mn^4+^. In the case of the B6TFO studied here, which is fired in an oxidising atmosphere, with no donor dopants presence, we would expect any Mn^2+^ that is present at the synthesis temperature to convert back to Mn^3+^ and Mn^4+^, and for oxygen to diffuse back into the lattice to compensate. The activation energy for an oxygen vacancy to diffuse in PKs is approximately 0.7 eV, while that for a *B*-site cation is between 10 and 15 eV^47^. Hence, we would expect that the oxygen stoichiometry in the system would easily normalise to compensate for any change in Mn oxidation state, but the cations would tend to stay locked in the positions determined during the film annealing process. The direct determination of Mn oxidation state in this system is an aspect of considerable interest because of its potential importance for magnetic properties, and will be the subject of future study.

The results from the localised compositional analysis at defects agrees with these observations. Region A in Fig. 5(a), for example, is a short region (seven octahedra) of PK units that is both adjacent to, and bounded on each edge by, (Bi_2_O_2_)^2+^. It would be reasonable to expect this region to be under greater compressive stress as a consequence, and indeed there is no Mn and very little Fe in this region. The localised 6-layer regions between OPBs are also richer in magnetic cations (especially Mn) in their *C* PK layers than the average 5-layer structure. Again, this would be expected from the non-linear energy model expounded above.

### Cation Partitioning and Magnetic Properties

The *B*-site magnetic cation content (47%) for disordered B6TFMO is above the percolation threshold (31% of magnetic sites)^42^ to permit NN coupling interactions. However, at the threshold, the number of NN super-exchange interactions is often too low to allow for RT magnetisation. An increase in the percentage of magnetic cations above the percolation threshold at the *C B*-sites (>8.5% on average and greater in the region of OPBs) would be expected to lead to an increase in the magnetic ordering temperature^4^. The DFT calculations performed by Birenbaum and Ederer^31^ have predicted the magnetic coupling strengths between NN and NNN interactions for the m = 4 BTFO system and shown that the strength of NN couplings for magnetic cations located within inner sites (*J*_NN_ ~43 to 46 meV) is more than twice that for adjacent inner to outer site (*J*_NN_ ~12 to 20 meV) NN coupling interactions, stressing the potential importance of the site-site interactions in the inner PK layers in determining magnetic behaviour. They also predict that in this system (with Fe^3+^ as the only magnetic cation), while for NN couplings, AFM coupling has lower energy than FM, (as would be expected from the Goodenough-Kanamori (GK) rules)^32^, there is little energy difference between the AFM and FM orientations for NNN and greater interactions. Moving to the possibilities within the B6TFMO system, according to the GK rules, Fe^3+^-O-Mn^4+^ or Mn^3+^-O-Mn^4+^ interactions are expected to show FM coupling via double-exchange mechanisms^33^. Furthermore, FM coupling of Mn^3+^-O-Mn^3+^ is also possible via semicovalent exchange *e.g*. for the longer Mn^3+^-O-Mn^3+^ bonds arranged perpendicular to the (100) planes in the perovskite-type manganites^33^. A decrease in the *c*/*a* ratio of the Aurivillius structure^31^ (and an increase in the in-plane Mn^3+^-O-Mn^3+^ bond lengths) as a result of increased partitioning of the larger magnetic cations to the *C* PK layers may lead to an increase in FM semicovalent interactions (see Supplementary Information). Hence, magnetic cation partitioning to the *C* sites in B6TFMO could be significant in permitting pathways to long-range RT magnetic order through an increase in the probability of potentially-ferromagnetic Fe-O-Mn or Mn-O-Mn NN interactions in the *C* and *I* layers, which are in any case going to be more important in mediating FM orientations than the *O* layers^31^. We can estimate the magnitude of this effect by using the relative proportions of the magnetic ions on the B-sites in the *C* layer. The probability of there being a potentially-ferromagnetic Fe-O-Mn or Mn-O-Mn NN interaction for an ion sitting in a *C* layer B-site is given by (see Supplementary Information):

3 $${P}_{F}=2{P}_{Fe}^{C}{P}_{Mn}^{C}+\,{P}_{Mn}^{C}{P}_{Mn}^{C}$$

where ${P}_{X}^{C}$ is the probability of finding the ion X on a *C* layer B-site.

We find that *P*_*F*_ = 0.1 for a B6TFO lattice with randomly distributed Fe and Mn ions, but is increased by 67% to *P*_*F*_ = 0.17 for the partitioned proportions reported in sample A and by 88% to *P*_*F*_ = 0.19 for the proportions reported in sample B. The corresponding increase for the *I* layer averages 50% for the two samples. It seems likely that this will have a positive effect on the percolation of magnetic spins in the system, and thus the probability of large-scale ferromagnetic effects emerging. We note that in this system, magnetic percolation is likely to take place through the spins on the Fe^3+^, Mn^3+^ and Mn^4+^ ions in both the *C* and *I* layers, with the potential for coupling both within and between the layers. Although the calculation of the percolation thresholds for this system are complex and beyond the scope of this work, we note that a value of *P*_*F*_ = 0.19 is of the same order as the percolation threshold that has been calculated for 2D and 3D systems when higher than NN interactions are considered^49, 50^.

It is recognized that in complex structures such as this, it would be interesting to have 3D compositional information, and atomic resolution imaging along other zone axes will be the subject of further work. The potential effects of defects such as stacking faults and OPBs may also be important in affecting the distribution of the magnetic cations. We note from this work that the Mn content of the lattice is depleted in the immediate region of an OPB, and therefore must be increased to some extent in the rest of the lattice. Hence, a high density of OPBs, as has been observed in some Aurivillius systems^36, 38^ and can be deliberately introduced through non-stoichiometry^51^, may be effective in increasing the degree of magnetic spin percolation, although more work is required to investigate this. Further work, perhaps using EPR, is also needed to determine the valence states of the magnetic ions in this B6TFMO system and this, coupled with more detailed theoretical studies should help engender a better understanding of the ferromagnetism that has been observed experimentally.

## Conclusions

We have shown that aberration corrected HAADF-STEM combined with EDX and EELS is capable of distinguishing between Ti/Mn/Fe of similar *Z* in a closely-packed material at the atomic-scale and this has provided clear atomic-scale demonstration of magnetic cation partitioning between the perovskite layers in in a multiferroic Aurivillius system, with some further localisation due to defects. In our samples, we have observed a strong preference for Mn to partition to the central PK layers, but only a slight tendency for the Fe to partition. The clear preference for Mn cations to partition into the *C* PK layers of B6TFMO is attributed to a combination of relieving the compressive stress at the (Bi_2_O_2_)^2+^/outer PK layer interface and to facilitate preferential occupation of the outer sites with cations having a larger effective electronic charge (Ti^4+^). A model has been presented to explain this partitioning based on the partial reduction of Mn^3+^ to the significantly-larger Mn^2+^ ion at the film synthesis temperature, an effect which is not expected to occur for Fe^3+^ in oxidizing conditions at this temperature. A Boltzmann-distribution-based model has also been presented that accurately describes the cation partitioning profiles, and indicates that the driving energy for Mn partitioning is ca 10x greater than that for Fe. The magnetic cation partitioning results in a notable increase in *B*-site magnetic cation composition from ~47% (~13%Mn) when considering a disordered structure to >54% (>19%Mn) with the observed partitioning at the *C* sites. We noted in the introduction that there is contradictory evidence for Fe ion partitioning ion the BTFO system, and this may well be due to differences in sample preparation methodologies (synthesis temperatures, atmospheres etc). Such differences could be expected to change the degree to which Fe^3+^ will reduce to Fe^2+^ during synthesis, and according to the model presented here, this would change the degree to which it would partition. Higher synthesis temperatures and/or more reducing firing atmospheres would have the effect of increasing the amount of Fe^2+^ during synthesis. Previous modelling of cation partitioning in the Aurivillius system^31, 42^ has been undertaken at room temperature, while the cation distribution necessarily takes place at the oxide synthesis temperature. We suggest that consideration of the cation oxidation states and lattice strains at these much higher temperatures may be a fruitful area for future theoretical research on cation partitioning in this system, and this insight may aid the experimental synthesis of materials in which higher magnetic spin percolation densities can be obtained.

Atomic-resolution chemical mapping reveals that the atomic structure at the defect regions deviates from stoichiometric composition and that disruptions to the lattice by e.g. OPBs results in further partitioning >59% (>20%Mn) of magnetic cations to the *C* PK layers of B6TFMO. The consequences and control of defect densities in this system may well be important in determining magnetic properties and requires further study. We have noted that the partitioning of Mn into the central PK layers will increase the probability of the potentially-ferromagnetic Fe-Mn and Mn-Mn nearest neighbour (NN) interactions. We calculate that this will be a nearly 90% increase in the central layer for the proportions observed here, and correspondingly reduce the probability of antiferromagnetic Fe-Fe NN interactions. We feel that these observations should strongly encourage further theoretical studies of this type of system, which hitherto^31^ have not considered the consequences of including Mn as a magnetic ion. Further experimental work is also required to determine the oxidation states of the Mn in this system.

## Methods

B6TFMO-A and B6TFMO-B were synthesized at separate times and in different growth runs, on *c*-sapphire substrates by liquid injection chemical vapour deposition (LI-CVD) methods^11^ and post-annealed at 850 °C (1123 K). The average stoichiometries, as determined by HR-SEM (high resolution scanning electron microscopy) with EDX, were Bi_6_Ti_3.04_Fe_1.42_Mn_0.54_O_18_ (B6TFMO-A) and Bi_6_Ti_2.99_Fe_1.46_Mn_0.55_O_18_ (B6TFMO-B). Analysis of B6TFMO A and B6TFMO B (over sample areas of 25 × 25 µm^2^ up to 1.2 × 1.2 mm^2^) was conducted using the FEI Helios Nanolab HR-SEM mode and EDX equipped with an X-Max 80 detector and AZTec analysis software from Oxford Instruments. Cross-sections of the B6TFMO films were prepared using a FEI Dual Beam Helios NanoLab 600i Focused Ion Beam (FIB) (final thinning @ 93 pA, 3 kV, final polish 2 kV, 28 pA) and were mounted on TEM grids. Thin coatings of Au were deposited on the sample surfaces to avoid possible surface charging effects. The samples were cleaned using a Fischione 1020 plasma cleaner prior to STEM imaging and EDX/EELS analysis. Imaging and analysis was performed on a NION UltraSTEM 200 operating at 200 kv. EDX analysis was performed using Bruker 100 mm^2^ windowless EDX detector. EDX acquisition and analysis was performed using Bruker Esprit 2.0. A Gatan Enfinium and GMS 2.0 was used for EELS acquisition and analysis. Energy filtered images acquired at 300 kV on an FEI Titan TEM with Gatan Tridiem Energy Filtering system demonstrated that thicknesses of the regions used for imaging were <35 nm. Further experimental details of the microscopy are given in the Supplementary Information. Images were taken along the [110] crystallographic zone axis. The EDX signal intensities for Ti, Fe and Mn were used as a proxy for the relative proportions of each atom on each of the five PK layers in the structure, normalized to 100% B-site occupancy. It has been noted^52, 53^ that inelastic scattering of electrons can lead to substantial differences between EDX measurements of composition and actual values. The inelastic scattering is proportional to the atomic numbers *Z* of the elements concerned, increases with sample thickness and is dependent upon the degree of electron channeling. In these measurements, the *Z* values of the atoms concerned are similar (*Z*_*Ti*_ = 22, *Z*_*Mn*_ = 25 and *Z*_*Fe*_ = 26), so any corrections for inelastic scattering would also be expected to be similar. In addition, it is the variation in the relative proportions of the magnetic ions across the PK layers that are of greatest interest in this work, and Fe and Mn differ only by one electron. The measurements were only taken along the [110] zone axis in each sample, so sample-to-sample effects due to channeling variations do not need to be taken into account. In addition, the sample was <35 nm thick and EDX intensity data was collected from, and averaged across, several different regions of different thicknesses of each sample. For these reasons, we believe that it is a fair assumption that the EDX intensities represent a good proxy for the relative atomic proportions of each atom. The fact that two unrelated samples of different thicknesses gave similar results, also gives confidence that this assumption is fair. A degree of signal-to-noise improvement was obtained by summing the EDX signal information for each ion along each layer for a total length of about 48 nm (or ca 360 unit cell lengths) for sample A and for 18 nm (or ca135 unit cells lengths) for sample B (see the green lines in Supplementary Figure S4(a and b)). This summation had the effect of integrating-out spatial noise.

## Electronic supplementary material

Supplementary Information
