# Supplementary material for: Direct atomic scale determination of magnetic ion partition in a room temperature multiferroic material
Source: Sci Rep. 2017 May 11;7:1737. doi: 10.1038/s41598-017-01902-1 (PMC5431865; doi:10.1038/s41598-017-01902-1)
Supplement: Supplementary file 1 — Supplementary Information [file 41598_2017_1902_MOESM1_ESM.pdf]

Supplementary Information for

Direct atomic scale determination of magnetic ion partition in a room temperature multiferroic material

*Lynette Keeney[1], Clive Downing[2], Michael Schmidt[1], Martyn E. Pemble[1, 3], Valeria Nicolosi[2] and Roger W. Whatmore[3, 4]**

1. Tyndall National Institute, University College Cork, 'Lee Maltings', Dyke Parade, Cork, Ireland.
2. School of Chemistry, CRANN, AMBER, Trinity College Dublin, Dublin 2, Ireland.
3. Department of Chemistry, University College Cork, Cork, Ireland.
4. Department of Materials, Faculty of Engineering, Imperial College London, London, SW7 2AZ, United Kingdom.

*Corresponding author. E-mail: r.whatmore@imperial.ac.uk

Microscopy Experimental Details

HAADF imaging was performed using a 27 mrad half-angle probe, the detector collection angle was about 99 – 200 mrad half-angle.

The EDX data was acquired and processed using Bruker Esprit 2.0, data collection was performed using a probe current ~130pA, a step size of 31pm and a dwell time of 4.1ms over a single frame. Each map of the integrated intensity of an element was then averaged using the Bruker software, lowering pixel resolution and integrating neighbouring pixels to increase signal to noise. This was reasonable as the step size was small and there was an amount of oversampling so each atomic column has several pixels.

The EELS data was acquired separately to the EDX and the data was collected and processed using Gatan Digital Micrograph. To produce the maps energy windows were placed over the Ti L3 peak 455-460eV, Mn L3 peak 638-642 and Fe L3 peak 705-711 with a power-law background subtracted before each edge. RGB colour maps were generated with an 8-bit pixel-depth using the colour mix feature in Digital Micrograph.

Supplementary Figure S1 (a) and (b) Atomic resolution HAADF-STEM images for B6TFMO of differing magnification illustrating that the majority of the Aurivillius phase is of the m=5 structure.

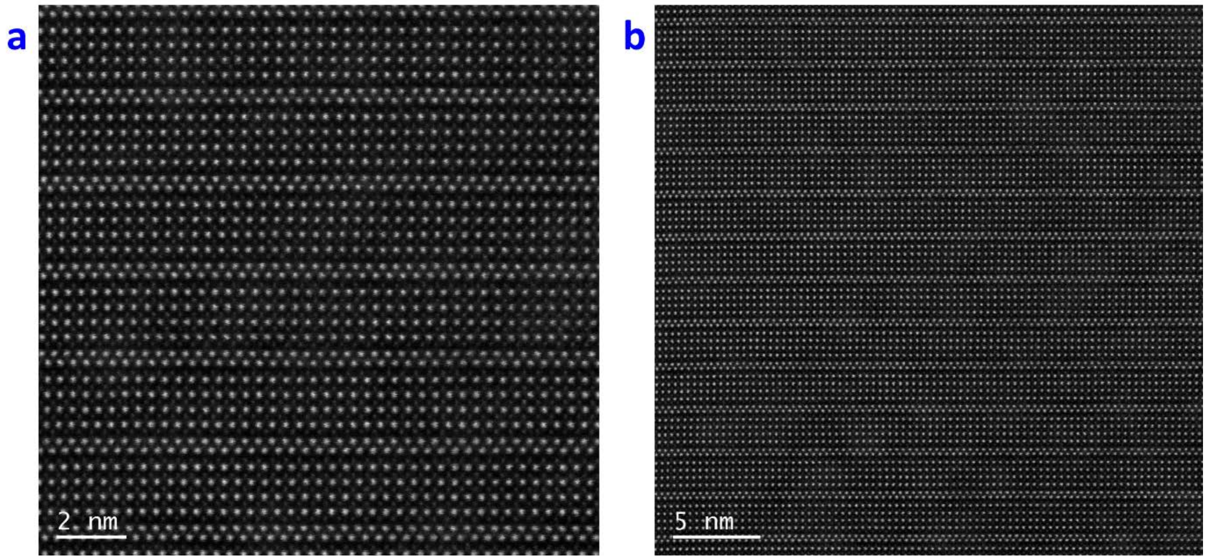

Supplementary Figure S2. Representative atomic resolution chemical mapping images for sample B6TFMO-B in a region of the crystallite without out of phase boundaries (OPBs) / stacking faults. **(a)** annular dark-field image and **(b)** to **(f)** corresponding EDX chemical composition maps: **(b)** overlay map showing annular dark-field image (white), Bi (green), Ti (blue), Mn (pink), Fe (red), **(c)** Bi map, **(d)** Ti map, **(e)** Mn map, **(f)** Fe map. **(g)** EELS chemical composition maps of a different region of B6TFMO-B (refer to **Supplementary Figure S3 (a)** for corresponding HAADF STEM image) with Ti in green and Mn in blue.

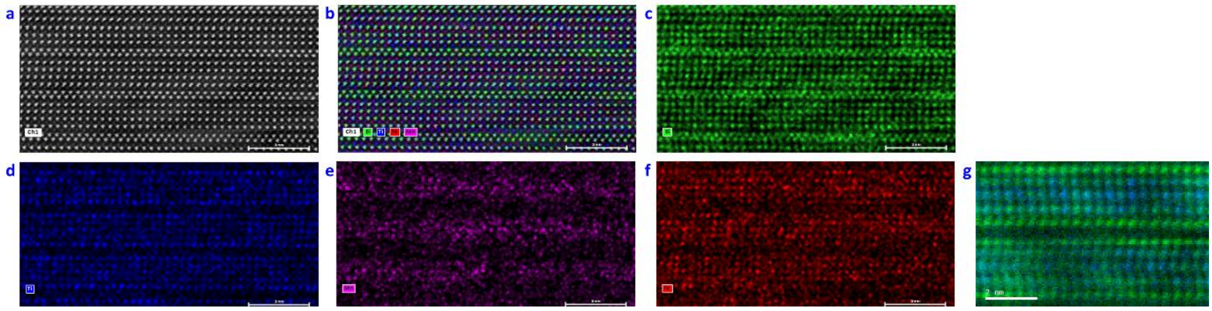

Supplementary Figure S3. Representative atomic resolution EELS images for B6TFMO samples. **(a)**, **(e)**, **(i)**, **(m)** are HAADF images, **(b)**, **(f)**, **(j)**, **(n)** are Ti maps, **(c)**, **(g)**, **(k)**, **(o)** are Mn maps, **(d)**, **(h)**, **(l)**, **(p)** are Fe maps. **(a)** to **(d)**: images for sample B6TFMO-A in a region of the crystallite without defects. **(e)** to **(h)**: images for sample B6TFMO-B in a region of the crystallite without defects. **(i)** to **(l)**: images for sample B6TFMO-A in a region of the crystallite displaying OPB defects. **(m)** to **(p)**: images for sample B6TFMO-B in a region of the crystallite displaying OPB defects.

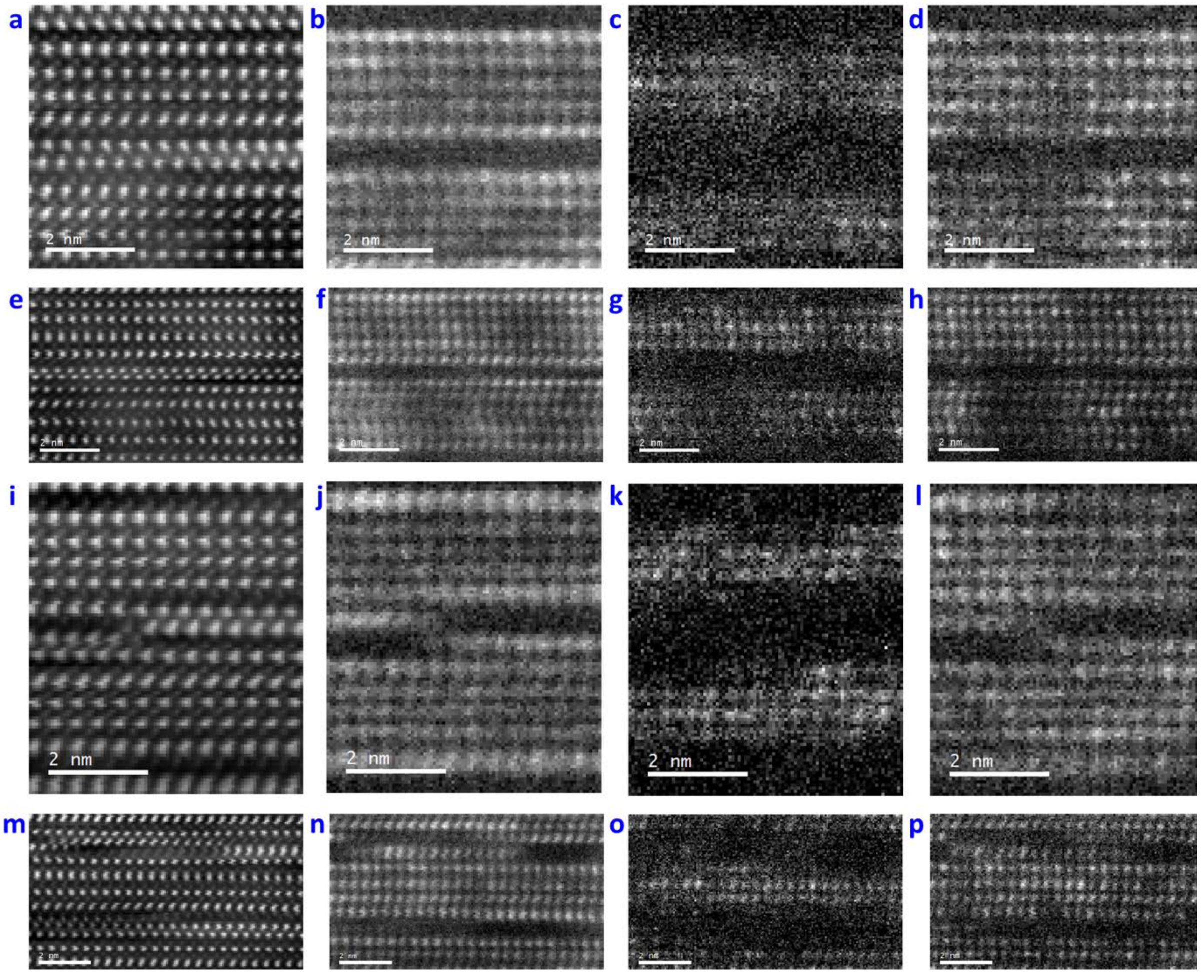

Supplementary Figure S4 a and S4b: Chemical mapping images for **(a)** Sample A and **(b)** Sample B illustrating the lines (shown in green on each image) along which the relative proportions of the Ti, Fe and Mn proportions were averaged to get the average relative *B*-site proportions shown in **Supplementary Table S1**.

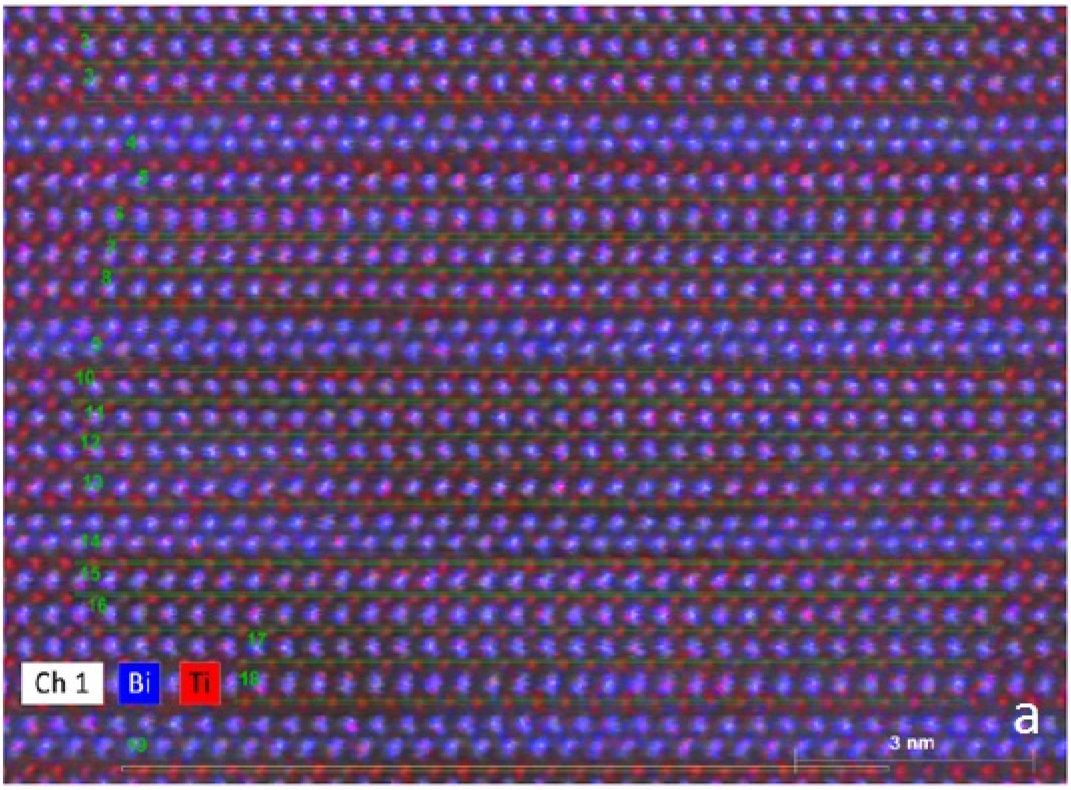

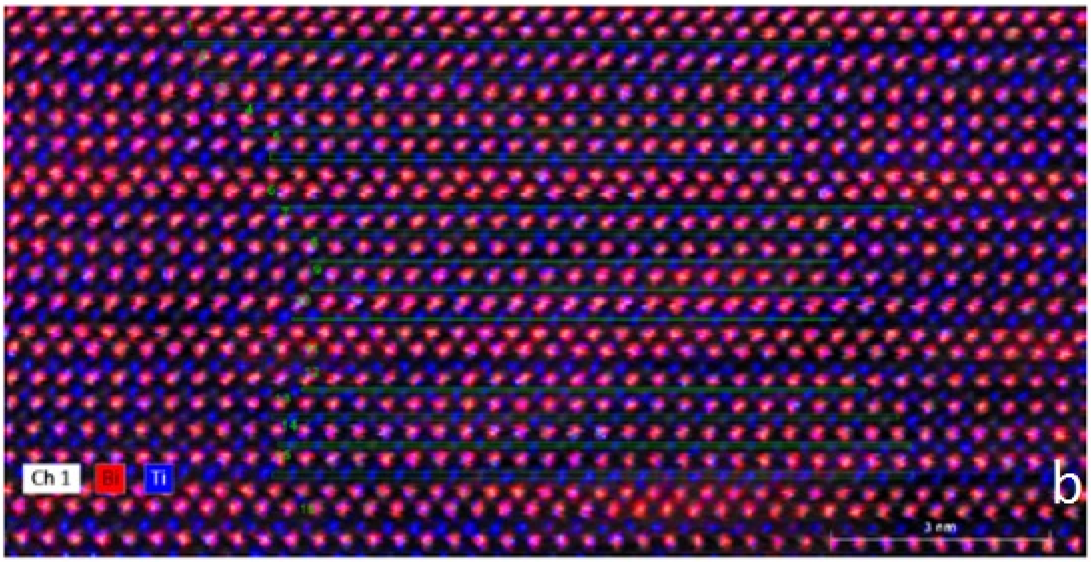

Supplementary Table S1: The average relative *B*-site proportions (expressed in terms of percentage of *B*-site occupancy) of Ti, Mn and Fe at the *O*, *M* and *C* layers of samples B6TFMO-A and B6TFMO-B. In this table the proportion of cations at the O (outer), M (intermediate) and C (centre) PK layers of the five-layered Aurivillius structure for the samples are compared with samples of the same average composition in which the ions are randomly distributed. Note that the data presented is for regions without OPB defects / stacking faults and was quantified by taking horizontal line profiles of the EDX data across the B-sites of the PK layers over three half-unit cells analysed.

	***B*-site content**			
**B6TFMO Sample A**	**%Ti**	**%Mn**	**%Fe**	**% Magnetic cations**
Average composition	55	13	32	45
O	66	4	30	34
M	48	19	33	52
C	46	19	35	54
	***B*-site content**			
**B6TFMO Sample B**	**%Ti**	**%Mn**	**%Fe**	**% Magnetic cations**
Average composition	52	13	35	48
O	61	7	32	39
M	48	15	38	53
C	44	21	35	56

Supplementary Table S2: Ionic Radii (6-fold coordination) for B-site cations[1]

Cation	Ionic radius (Å)
Ti^{4+}	0.605
Fe^{3+}	0.645
Fe^{2+}	0.77
Mn^{3+}	0.65
Mn^{2+}	0.82

<u>Calculation of Shannon Site Distortion parameter [1]:</u>

$$\Delta_n = \frac{1}{n}\sum_i \left\{\frac{r_i - r_m}{r_m}\right\}^2 \times 1000 \qquad (1)$$

Where r_i and r_m are the individual and mean bond lengths respectively and the sum is taken over all the bonds (in this case n = 6).

Giddings *et al.*[2] determined that Δ_6 is 5.2 and 5.6 for the outer and inner octahedral respectively at 295K in their *m*=4 system. There is insufficient difference between these figures to drive cation partitioning, but at 970K (much closer to the compound synthesis temperature) the numbers are 6.17 and 0.75 respectively, providing a clear driving mechanism.

Energetics for the reduction of Mn^{3+} and Fe^{3+} at 1100 K:

If we consider the two reduction reactions:

$$6Mn_2O_3 \rightarrow 4Mn_3O_4 + O_2 \qquad \textbf{(2)}$$

and

$$6Fe_2O_3 \rightarrow 4Fe_3O_4 + O2 \qquad \textbf{(3)}$$

We can calculate the standard enthalpy (ΔH_f) and Gibbs free energy of the reaction at the synthesis temperature of 1100K (ΔG) using literature thermodynamic data[3].

For reaction **(2)**: ΔH_f = 202 kJ/mol and at 1100 K ΔG = -7.63 kJ/mol

For reaction **(3)**: ΔH_f = 471.6 kJ/mol and at 1100 K ΔG = 179 kJ/mol

Accordingly, it is much easier to reduce Mn^{3+} than Fe^{3+} at the B6TFMO synthesis temperature, and as ΔG is negative we expect the reaction to proceed forwards. If the thermodynamics is similar in the context of the Aurivillius phase environment, this means that a substantial part of the Mn in the system will be present as Mn^{2+}. For iron ΔG is positive, which means that there will be no Fe^{2+}.

Boltzmann equation to model the expected distribution of magnetic cations within PK layers:

The Gaussian, or Normal distribution model can be applied to model cation distribution. This is described by:

$$C(x) = \frac{1}{\sqrt{2\pi\sigma^2}} \exp\left\{\frac{-(x-\mu)^2}{2\sigma^2}\right\} \qquad \textbf{(4)}$$

where μ = value of x for which *C*(x) (the concentration of an ion at a point x in the structure) is a maximum and σ = standard deviation.

The statistical distribution of particles between states of different energy *ΔE* is given by the Boltzmann Distribution therefore we can use the Boltzmann equation to model the expected distribution of the Mn and Fe within the three perovskite layers. We can write the concentration of an ion *C(x)* at a point x in the structure (where x=0 for the Centre layer, x=0.4α for the Intermediate layer and x=0.8α for the Outer layer and α~1nm, based on the published 5 layer structure[4]) as:

$$C(x) = C(0)exp\left\{\frac{-\Delta E(x)}{kT}\right\} \qquad \textbf{(5)}$$

Where *k* is Boltzmann's constant, *T* is the absolute temperature and Δ*E(x)* is the energy difference between the ion sitting in the layer and the same ion sitting in the centre layer. We can model Δ*E* as a quadratic function of x, representing the fact that there is a big difference in energy between an ion sitting on the outer later, relative to the intermediate layer, but the energy difference between the intermediate and centre layers is not so great:

$$\Delta E(x) = bx^2 \qquad \textbf{(6)}$$

Now, we find that:

$$C(x) = C(0)exp\left\{\frac{-bx^2}{2\sigma}\right\} \quad \textbf{(7)}$$

Which is a Gaussian function (see **Equation (4)**), with $\sigma = \sqrt{\frac{kT}{2b}}$, or $b = \frac{kT}{2\sigma^2}$ **(8)**

Semicovalent ferromagnetic exchange interactions in perovskite-type manganates:

The original paper by Goodenough[5] on the [La,M(II)]MnO_3 points out that Mn^{3+}-O-Mn^{3+} sequences can show ferromagnetic coupling, depending upon the degree of covalency in the bonds. In the case of the perovskite rare earth manganites, this depends on the radius of the A-site cation. In the case of La^{3+}, the ionic radius is 1.18 Å in 8-fold coordination by oxygen, compared with 1.11 Å in Bi^{3+}. The much later paper by Zhou & Goodenough[6] indicates that in the rare earth manganate perovskites, the smaller the A-site cation, the stronger the Jahn-Teller distortion, and the lower the antiferromagnetic Nèel temperature. In this report, $GdMnO_3$ has the optimal orbital mixing for ferromagnetic coupling. Bi^{3+} is much more similar in size to Gd^{3+} (1.11 Å) than La^{3+}, and the Nèel temperature for $GdMnO_3$ is <50 K, with a high JT transition temperature (1400 K). On these grounds, we might reasonably expect a significant proportion of Mn^{3+}-O-Mn^{3+} sequences to be ferromagnetic in character in B6TFMO.

Probability of Bonds being Potentially-Ferromagnetic Sequences

We wish to know the probability of a M-O-M bond sequence in the *Central* perovskite layer (**Supplementary Figure S5**) being a potentially-ferromagnetic Fe-O-Mn or Mn-O-Mn sequence. We define the probability of finding a particular ion on the B-site of a particular layer as shown in **Supplementary Table S3**.

Supplementary Figure S5: Labelling of planes in the 5-layer Aurivillius structure.

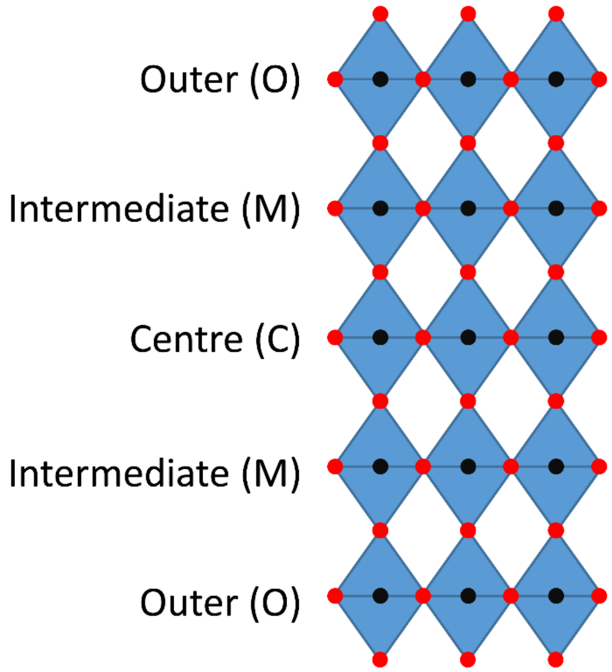

Supplementary Table S3: Probability of finding an ion on any particular B-site in the structure

Layer	Centre	Intermediate	Outer
Iron	P_{Fe}^{C}	P_{Fe}^{I}	P_{Fe}^{O}
Manganese	P_{Mn}^{C}	P_{Mn}^{I}	P_{Mn}^{O}

(Assume here that the structure is symmetrical about the Centre layer)

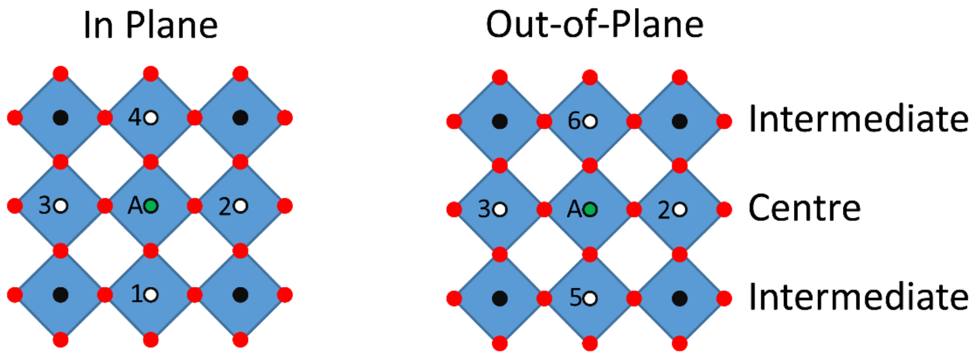

Supplementary Figure S6: The site-numbering scheme used to compute the probabilities of M-O-M bonds in the Central layer being potentially-ferromagnetic Fe-O-Mn or Mn-O-Mn sequences.

Starting on a B-site, labelled “A” in the Centre layer as shown in **Supplementary Figure S6a** below, the probability of that being iron is P_{Fe}^{C} . We can get the probability of this iron atom being linked through oxygen to a manganese on any of the sites labelled 1 to 4 (in the centre layer) by multiplying this by the probability of the site in question being occupied by Mn, which is P_{Mn}^{C}, which is:

$$P_{Fe-O-Mn}^{C} = P_{Fe}^{C} P_{Mn}^{C} \qquad \textbf{(9)}$$

Similarly, if “A” is occupied by Mn, we can calculate the probability of the bond being a Mn-O-Fe sequence as:

$$P_{Mn-O-Fe}^{C} = P_{Mn}^{C} P_{Fe}^{C} \qquad \textbf{(10)}$$

Also, a M-O-M bond in the Centre layer being an Mn-O-Mn sequence is:

$$P_{Mn-O-Mn}^{C} = P_{Mn}^{C} P_{Mn}^{C} \qquad \textbf{(11)}$$

Hence, the probability of there being a potentially-ferromagnetic Fe-O-Mn or Mn-O-Mn NN interaction for any ion sitting in a *C* layer B-site is given by the sum of **Equations (9), (10)** and **(11)**:

$$P_F = 2P_{Fe}^{C} P_{Mn}^{C} + P_{Mn}^{C} P_{Mn}^{C} \qquad \textbf{(12)}$$

Based on the average compositions of the single grains as determined by NION, the probabilities of site occupancy for randomly distributed ions would be:

	Centre	Intermediate	Outer
Ti	0.53	0.53	0.53
Fe	0.34	0.34	0.34
Mn	0.13	0.13	0.13

For this, P_F = 0.1

For sample A, averaging the compositions of the Intermediate and Outer Layers, we have

	Centre	Intermediate	Outer
Ti	0.46	0.48	0.66
Fe	0.35	0.33	0.30
Mn	0.19	0.19	0.04

For this, $P_F = 0.17$

For sample B, averaging the compositions of the Intermediate and Outer Layers, we have

	Centre	Intermediate	Outer
Ti	0.44	0.48	0.61
Fe	0.35	0.38	0.32
Mn	0.21	0.15	0.07

For this, $P_F = 0.19$

References:

1 Shannon, R. D., Revised effective ionic radii and systematic studies of interatomic distances in halides and chalcogenides. *Acta Crystallographica Section A* **32**, 751-767 (1976).

2 Giddings, A. T. , *et al.*, Synthesis, structure and characterisation of the n=4 Aurivillius phase Bi5Ti3CrO15, *Journal of Solid State Chemistry* **184**, 252-263 (2011).

3 Fritsch, S. and Navrotsky A., Thermodynamic Properties of Manganese Oxides. *J. Am. Ceram. Soc.* **79**, 1761-1768 (1996).

4 GarcÍA-Guaderrama, M., Fuentes-Montero, L., Rodriguez, A. and Fuentes, L. Structural Characterization of Bi6Ti3Fe2O18 Obtained by Molten Salt Synthesis, *Integrated Ferroelectrics* **83**, 41-47 (2006).

5 Goodenough, J. B., Theory of the Role of Covalence in the Perovskite-Type Manganites [La,M(II)]MnO3, *Physical Review* **100**, 564-573 (1955).

6 Zhou, J. S. and Goodenough, J.B., Unusual Evolution of the Magnetic Interactions versus Structural Distortions in REMnO3 Perovskites, *Phys. Rev. Lett.* **96**, 247202 (2006).
